# T cell kinetics reveal expansion of distinct lung T cell subsets in acute versus in resolved influenza virus infection

**DOI:** 10.3389/fimmu.2022.949299

**Published:** 2022-10-06

**Authors:** Malin Eriksson, Susanne Nylén, Kjell-Olov Grönvik

**Affiliations:** ^1^ Department of Microbiology, Tumor and Cell Biology, Karolinska Institutet, Stockholm, Sweden; ^2^ Department of Microbiology, National Veterinary Institute, Uppsala, Sweden; ^3^ Uppsala Immunobiology Lab, Uppsala, Sweden

**Keywords:** influenza virus, resident T cells, Trm, CD69, CD103, CD49a

## Abstract

Influenza virus infection is restricted to airway-associated tissues and elicits both cellular and humoral responses ultimately resulting in generation of memory cells able to initiate a rapid immune response against re-infections. Resident memory T cells confer protection at the site of infection where lung-resident memory T cells are important for protecting the host against homologous and heterologous influenza virus infections. Mapping kinetics of local and systemic T cell memory formation is needed to better understand the role different T cells have in viral control and protection. After infecting BALB/c mice with influenza virus strain A/Puerto Rico/8/1934 H1N1 the main proportion of activated T cells and B cells expressing the early activation marker CD69 was detected in lungs and lung-draining mediastinal lymph nodes. Increased frequencies of activated cells were also observed in the peripheral lymphoid organs spleen, inguinal lymph nodes and mesenteric lymph nodes. Likewise, antigen-specific T cells were most abundant in lungs and mediastinal lymph nodes but present in all organs studied. CD8^+^CD103^-^CD49a^+^ lung-resident T cells expanded simultaneously with timing of viral clearance whereas CD8^+^CD103^+^CD49a^+^ lung-resident T cells was the most abundant subset after resolution of infection and antigen-specific, lung-resident T cells were detected up to seven months after infection. In conclusion, the results in this detailed kinetic study demonstrate that influenza virus infection elicits adaptive immune responses mainly in respiratory tract-associated tissues and that distinct subsets of lung-resident T cells expand at different time points during infection. These findings contribute to the understanding of the adaptive immune response locally and systemically following influenza virus infection and call for further studies on the roles of the lung-resident T cell subsets.

## Introduction

Despite existing vaccines and extensive research, influenza virus continues to pose a global health threat and approximately 291000 – 645000 human influenza A virus-related respiratory deaths are estimated to occur annually (1). The infection is normally restricted to the respiratory tract resulting in symptoms including cough, fatigue, fever and malaise (2, 3). After viral entry, the surface glycoprotein hemagglutinin binds to α2,3-linked sialic acid receptors on ciliated airway epithelial cells (4). Once inside the epithelial cell, the virus uses the host machinery to produce viral progeny which is released from the cell, a process facilitated by the other influenza virus surface glycoprotein neuraminidase (5). Dendritic cells (DCs) in the lungs acquire influenza virus antigens from infected airway epithelial cells or by direct infection of the DCs (6–8). The DCs then migrate to lung-draining mediastinal lymph nodes (MLNs) and to spleen *via* afferent lymphatic vessels where they prime CD4^+^ and CD8^+^ T cells 2 – 3 days *post* infection (dpi) by establishing stable contacts resulting in activation of antigen-specific T cells (9–14). CD69 is expressed within hours after engagement of the antigen-binding receptor with its cognate antigen and is used as a marker of early activation of T cells, B cells and NK cells (15–17). After up to 48 hours of interaction with DCs the T cells detach from the DCs, start their clonal expansion but remain in the MLNs for another 48 hours before migrating to the lungs (9, 10). Natural infection with influenza virus elicits both B and T cell responses with subsequent development of humoral and cellular immunity after viral clearance (14). Unlike B cells, T cells recognize conserved influenza virus epitopes thus providing protection against heterosubtypic infections as well as potential protection against antigenic drift variants (18). Antigen-specific effector and memory T cells express CD44, indicative of antigen-experience, and populate lymphoid and non-lymphoid organs throughout the body (19–22). In contrast to CD69, CD44 is upregulated on T cells within days after activation (23).

Tissue-resident memory T cells (Trm) constitute heterogenous populations located in various tissues throughout the body e.g. skin, intestines, salivary glands and lungs (24, 25). Trm in the lungs are assigned to be of particular importance for conveying heterosubtypic immunity and provide local protection against invading pathogens by e.g. clearing influenza virus 36 – 48 hours faster after re-infection (18, 26). Both CD4^+^ and CD8^+^ Trm are established after influenza virus infection in mice and based on various surface markers different subsets can be distinguished (26–28). The following three subsets of CD8^+^CD69^+^ lung-resident memory T cells have been described based on surface expression of integrins CD49a and CD103: CD103^+^CD49a^-^ epithelial effector Trm (eeTrm), CD103^+^CD49a^+^ epithelial Trm (eTrm) and CD103^-^CD49a^+^ interstitial Trm (iTrm) (28). The subsets are proposed to reside at different locations in the lung tissue with iTrm in the interstitium, eeTrm within the respiratory epithelium and eTrm in the respiratory epithelium in contact with the basement membrane (28). All subsets have effector functions including production of antiviral cytokines with CD103^+^CD49a^+^ Trm expressing the highest level and CD103^+^CD49a^-^ Trm the lowest level (29). Duration of local protection against heterosubtypic challenge is reported to decline at 15 weeks *post* priming and is depleted 4 – 5 months *post* immunization (18). In comparison, influenza virus-specific T cells in the spleen remain at least for two years after infection (30).

Apart from the lung-draining lymph nodes, priming of T cells occurs in the spleen after influenza virus infection. However, little is known about priming of T cells in other lymphoid organs and in this study, we sought to investigate whether an immune response against influenza virus was elicited in secondary lymphoid organs not draining the site of infection and its kinetics. Furthermore, the kinetics of different pulmonary Trm subsets after influenza virus infection has not been studied previously. Therefore, we performed a kinetic study of the B cell and T cell responses in lungs and lung-draining MLNs as well as in spleen, inguinal lymph nodes (ILNs) and mesenteric lymph nodes (MesLNs). We demonstrate that activation of the adaptive immune response with subsequent differentiation of antigen-specific effector memory T cells (Tem) occurs in lungs and MLNs and to a minor extent in the other organs studied. Furthermore, expansion of lung-resident CD8^+^CD103^-^CD49a^+^ T cells was observed simultaneously with viral clearance whereas CD103^+^CD49a^+^ T cells was the dominating subset early after resolution of infection but both subsets remained at 7 months *post* infection, and we therefore hypothesize on the biological significance of these findings.

## Materials and methods

### Mice and influenza virus infection

Female BALB/cAnNCrl mice aged 5 – 9 weeks bred and maintained under specific pathogen-free conditions at the animal facility of the National Veterinary Institute (SVA), Uppsala, Sweden were used. The mice were housed in groups of up to eight mice under climate-controlled conditions with a 12h:12h light-dark cycle in open-top conventional EU type 3 cages (dimensions 425 mm x 276 mm x 153 mm, floor area 820 cm^2^, Tecniplast) and fed R3 (Lantmännen, Stockholm, Sweden) and/or RM3A (P) (SDS, Essex, United Kingdom) *ad libitum*. The mice were infected intranasally with 2.5 x 10^4^ TCID_50_ influenza virus (strain A/Puerto Rico/8/1934 H1N1) (PR8) cultured in-house or with 2 x 10^3^ TCID_50_ PR8 purchased from Charles River (Charles River Laboratories, RRID : SCR_003792) in 25 µl PBS under light isoflurane anesthesia. The in-house PR8 was generated and purity confirmed as previously described (31). All animal experimental procedures were in accordance with EU directive 2010/63/EU and approved by the regional animal ethics committee of Uppsala, Sweden.

### *In vivo* treatment with anti-CD45.2 antibodies

200 μl PBS (SVA, Uppsala, Sweden) with 3 μg of FITC-conjugated antibodies against the alloantigen CD45.2, present on the majority of hematopoietic cells, was injected intravenously in the lateral tail vein of mice three minutes prior to euthanasia according to the protocol elaborated by Andersson et al. (32). Splenic cells and peripheral blood leucocytes were analyzed as controls of successful staining.

### Flow cytometry

**Figure 1:**
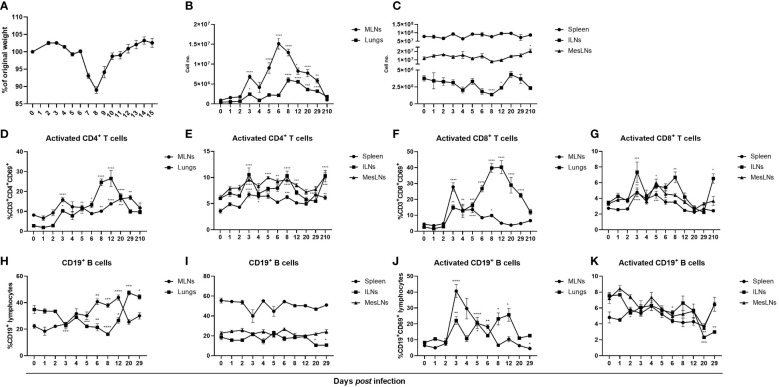
Influenza virus infection in mice results in a transient weight loss and activation and proliferation of B cells and T cells primarily in airway-associated tissues. BALB/c females aged 4 – 9 weeks old were infected with the mouse adapted H1N1 influenza A virus PR8 and monitored daily for changes in weight **(A)**. At indicated time points the mice were euthanized followed by collection of MLNs, lungs, spleen, ILNs and MesLNs and viable cells in single-cell suspensions were counted using trypan blue exclusion test **(B, C)** and analyzed with flow cytometry. Frequencies of activated helper T cells and cytotoxic T cells gated on CD3^+^ lymphocytes in MLNs, lungs **(D, F)**, spleen, ILNs and MesLNs **(E, G)**. Frequencies of CD19^+^ B cells gated on total lymphocytes **(H, I)** and activated CD19^+^ B cells **(J, K).** Data in A is representative weight data from one separate experiment. Data in figures B – K are composites of 1 – 4 separate experiments per time point with 3 – 10 mice per group. Data are shown as mean ± SEM. *p < 0.05, **p < 0.01, ***p < 0.001 and ****p < 0.0001.

At 1 – 210 days *post* infection the mice were euthanized by cervical dislocation and MLNs, lungs, spleens, ILNs and MesLNs were collected in 10 ml PBS (SVA, Uppsala, Sweden). Single-cell suspensions were prepared by passage through a metal mesh followed by repeated passages through a 23-gauge needle. To remove red blood cells, MLN, lung and splenic cells were lysed with ammonium-chloride-potassium (ACK) lysing buffer (SVA, Uppsala, Sweden) for 1 – 2 minutes and suspended in FACS buffer (PBS + 1% fetal calf serum). The number of viable cells were determined using Trypan blue (SVA, Uppsala, Sweden) exclusion test. The cells were stained for 30 minutes at +4°C with the following monoclonal antibodies diluted 1:50 in FACS buffer: PerCP hamster anti-mouse CD3e (145-2C11) (BD Biosciences Cat# 553067, RRID : AB_394599), V500 rat anti-mouse CD4 (RM4-5) (BD Biosciences Cat# 560782, RRID : AB_1937315)/FITC rat anti-mouse CD4 (GK1.5) (BD Biosciences Cat# 553729, RRID : AB_395013), APC rat anti-mouse CD8α (53-6.7) (BD Biosciences Cat# 553035, RRID : AB_398527), BV510 rat anti-mouse CD8α (53-6.7) (BD Biosciences Cat# 563068, RRID : AB_2687548), FITC rat anti-mouse CD8α (53-6.7) (BD Biosciences Cat# 553031, RRID : AB_394569), PE rat anti-mouse CD44 (IM7) (BD Biosciences Cat# 553134, RRID : AB_394649), FITC rat-anti mouse CD44 (IM7) (BD Biosciences Cat# 553133, RRID : AB_2076224), APC rat anti-mouse CD62L (MEL-14) (BD Biosciences Cat# 553152, RRID : AB_398533), APC-Cy7 rat anti-mouse CD62L (MEL-14) (BD Biosciences Cat# 560514, RRID : AB_10611861), V450 rat anti-mouse CD19 (1D3) (BD Biosciences Cat# 560375, RRID : AB_1645269), BV510 rat anti-mouse CD103 (M290) (BD Biosciences Cat# 563087, RRID : AB_2721775), PerCP-Cy5.5 hamster anti-rat/mouse CD49a (Ha31/8) (BD Biosciences Cat# 564862, RRID : AB_2734135), PerCP-Cy5.5 rat anti-mouse CD11a (2D7) (BD Biosciences Cat# 562809, RRID : AB_2737809), and PE-Cy7 hamster anti-mouse CD69 (H1.2F3) (BD Biosciences Cat# 552879, RRID : AB_394508), all from BD Biosciences (BD Biosciences, RRID : SCR_013311). To block unspecific binding purified rat anti-mouse CD16/CD32 (2.4G2) (BD Biosciences Cat# 553142, RRID : AB_394657) diluted 1:50, was added. For detection of antigen-specific T cells the cells were stained with tetramers before surface staining. The following reagents were obtained through the NIH Tetramer Core Facility: H-2K(d) influenza A NP_147 -155_, I-A(d) influenza A M_2 - 17_ tetramers and CLIP peptide-containing negative controls conjugated to BV421 and APC. For tetramer and CLIP-peptide containing controls, cells were stained with 4 µg/ml tetramer/CLIP in high-glucose DMEM supplemented with pyruvate, GlutaMAX™ and 2% fetal calf serum (Gibco) at +37.5°C for 75 minutes. After staining the cells were washed once, suspended in FACS-buffer and acquired on a BD FACSVerse™ and analyzed with FlowJo (FlowJo, RRID : SCR_008520) version 10. Fluorescence minus one (FMO) controls were used for gating. Gating strategy for activated T and B cells, CD44hi and Tem is shown in **Figure 1** in (**Table 1 in Supplementary Material**).

### Detection of viral RNA

Tissue specimens for PCR were taken from bone marrow, MLNs, lungs, ILNs, and spleen from mice infected intranasally with 10^4^ or 10^6^ TCID_50_ PR8 two days earlier. The qualitative determination of H1N1 PR8 influenza virus nucleic acid in the tissues was performed by the molecular diagnostic laboratory at the National Veterinary Institute, Uppsala, Sweden using an AgPath-ID one-step real-time transcriptase PCR (RT-PCR) kit (Applied Biosystems). Tissue samples were homogenized in a Tissue Lyser (Qiagen), and extraction of virus nucleic acid was done with Nordiag Magnatrix 8000+ extraction robot using a Viral NA kit (Nordiag). The following PCR primers were used: PaninflAForward, GGGTAGATAATCACTCACTGAGTG, PaninflAReverse, CTCTGATYTCAGTNGCATTCTG, and pan-influenza virus NP, 6-FAM–ATGGCGTCTCAAGGCACCAAACG–BHQ-1 (fluorescence-labeled probe with black hole quencher). Positive virus controls and negative Tris-EDTA (TE) buffer (SVA, Uppsala, Sweden) controls were used in the real-time RT-PCR.

### Statistical analysis

GraphPad Prism (GraphPad Prism, RRID : SCR_002798) version 9 was used to prepare the graphs and perform the statistical analysis. To test whether frequencies and cell numbers after infection were statistically significant from those of naïve mice, one-way ANOVA followed by Tukey's multiple comparisons test were performed. When variances of the populations were significantly different as determined by Bartlett’s test and Brown-Forsythe test the latter and Welch ANOVA tests were performed followed by Dunnett’s T3 multiple comparisons test. Data is shown as mean ± standard error of the mean (SEM) and stars indicate a statistically significant difference when compared with values obtained from uninfected mice (0 dpi). The following was used to denote statistical significance * = p< 0.05, ** = p< 0.01, *** = p< 0.001 and **** = p< 0.0001.

## Results

### Influenza virus infection results in activation of T cells and B cells in respiratory tract-associated tissues and peripheral lymphoid organs

To test the spread of the virus in lymphoid tissues and in lungs, groups of three BALB/c mice were infected intranasally with a sublethal dose or a lethal dose of PR8. At 48 hours *post* infection the Ct values indicated significant presence of viral RNA in a dose-dependent manner in MLNs and in lungs. No viral RNA was detected in spleen, ILNs or in bone marrow (**Table 1 in Supplementary Material**). Thus, the intranasal administration route mimics a natural infection *via* the upper airways where influenza virus is contained within susceptible tissues of the respiratory tract.

To study the adaptive immune response after influenza virus infection mice were infected intranasally with PR8 and euthanized at indicated time points up to 7 months *post* infection. The cardinal symptom is a dose-dependent transient weight loss which reaches its maximum at 7 – 10 dpi (**Figure 1A**) (3, 33). A first wave of cell accumulation was observed in lungs and lung-draining MLNs at 3 dpi followed by a second wave at 6 and 8 dpi in MLNs and lungs respectively (**Figure 1B**). In previous reports it was found that pulmonary inflammation persists at least three weeks after influenza virus infection which is in line with our finding that total cell numbers in MLNs and lungs were still increased at 29 dpi but had returned to baseline levels at seven months *post* infection (34). However, cell numbers in ILNs were significantly reduced days 8 and 12 *post* infection, possibly reflecting a migration of cells to the site of infection (**Figure 1C**). Using flow cytometry cell surface expression of the early activation marker CD69 was analyzed in T cells and in B cells. The infection triggered activation of both helper and cytotoxic T cells in MLNs, lungs, spleen and ILNs with the highest frequencies observed in MLNs and lungs (**Figures 1D–G**). In MLNs, lungs and spleen the numbers of activated T cells reflected the increased frequencies (**Figure 2 in Supplementary Material**). In MLNs, a biphasic activation of T helper cells was observed whereas in lungs the proportion of CD69-expressing T cells continued to increase until 12 dpi (**Figure 1D**). At seven months *post* infection the frequencies of CD69-expressing T helper cells in spleen, ILNs and MesLNs and cytotoxic T cells in ILNs were slightly increased (**Figures 1E, G** **)**. The frequency of CD19^+^ B cells decreased in lungs 5 – 8 dpi with the corresponding increase in MLNs that lasted until 12 dpi (**Figure 1H**). After 8 dpi the proportion of CD19^+^ B cells increased in lungs and remained increased until 29 dpi (**Figure 1H**). The proportion of CD19^+^ B cells returned to baseline in MLNs by day 20 *post* infection (**Figure 1H**). In spleen and ILNs a minor decrease in frequencies, but not numbers, of CD19^+^ B cells occurred at 20 and 29 dpi whereas the frequency remained unaltered in MesLNs (**Figure 1I**, **Figure 2 in Supplementary Material**). Activated CD19^+^ B cells increased substantially at 3 dpi and decreased until 12 dpi in MLNs but in lungs a minor increase in frequency was observed at 3 dpi and then a second increase was observed at 8 – 12 dpi (**Figure 1J**) whereas the frequencies decreased in MesLNs at 6, 8 and 20 dpi; and 2 and 29 dpi in ILNs (**Figure 1K**). The results demonstrate that influenza virus infection activates the adaptive immune response in lungs and lung-draining MLNs to a higher degree than in the peripheral lymphoid organs spleen, ILNs and MesLNs.

### Differentiation of antigen-specific T cells occurs locally at the site of infection and systemically after influenza virus infection

**Figure 2:**
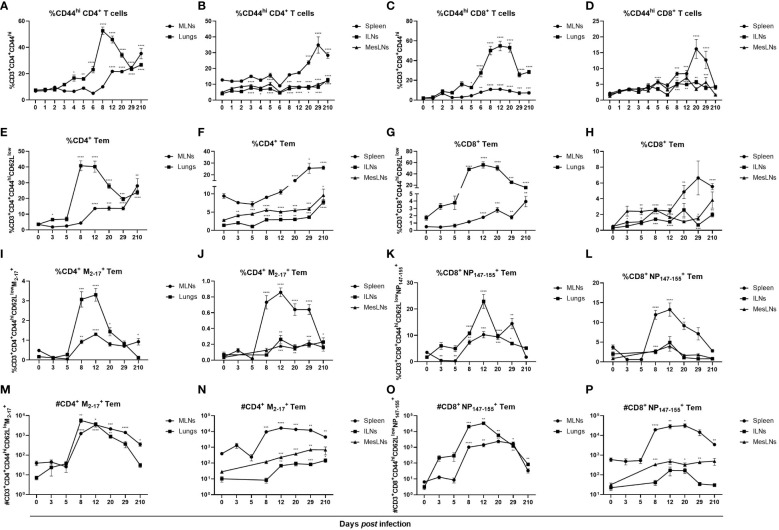
Influenza virus infection induced antigen-specific CD4^+^ and CD8^+^ Tem locally and systemically. Cells were stained with anti-CD44 antibodies to investigate the differentiation of effector T cells. CD44^hi^ cells gated on CD3^+^CD4^+^ or CD3^+^CD8^+^ T cells in MLNs, lungs **(A, C)**, spleen, ILNs and MesLNs **(B, D)**. To separate CD44^hi^CD62L^low^ Tem from CD44^hi^CD62L^hi^ central memory T cells in MLNs, lungs **(E, G),** spleen, ILNs and MesLNs **(F, H)** antibodies against the L-selectin CD62L was added to the flow cytometry panel. Frequencies of antigen-specific Tem were determined by staining with class II MHC tetramer M_2-17_ **(I, J)** and class I MHC tetramer NP_147-155_ **(K, L)**. Numbers of antigen-specific CD4^+^ Tem **(M, N)** and antigen-specific CD8^+^ Tem **(O, P)**. Data in figures A – D are composites of 1 – 4 separate experiments per time point with 3 – 10 mice per group. Data in figures E – P are composites of 2 – 3 separate experiments per time point with 4 – 10 mice per group. Data are shown as mean ± SEM. *p < 0.05, **p < 0.01, ***p < 0.001 and ****p < 0.0001.

Formation of effector cells in lungs after influenza virus infection has previously been demonstrated (33). Since an increase of activated T cells was observed in peripheral lymphoid organs not directly involved in the infection, we investigated whether antigen-specific effector T cells were also present. As enhanced frequencies of CD44-expressing T cells were observed in all organs investigated, apart from CD8^+^CD44^hi^ T cells in ILNs, at 29 dpi (**Figures 2A–D**) anti-CD62L antibodies were added to discriminate Tem (CD44^hi^ CD62L^low^) from central memory T cells (CD44^hi^ CD62L^hi^). Increased frequencies and numbers of Tem were observed in all organs investigated but were most increased in lungs with a peak at days 8 – 20 *post* infection (**Figures 2E–H**; **Figure 3 in Supplementary Material**). Tem populations in MLNs were significantly increased at 8 dpi and remained increased until 29 dpi (**Figures 2E, G**). In spleen, Tem populations did not increase until days 20 and 29 *post* infection (**Figures 2F, H**). In ILNs, frequencies of both CD4^+^ and CD8^+^ Tem were elevated at 8 dpi and remained increased until 29 dpi (**Figures 2F, H**). At 3 dpi CD4^+^ Tem and at 5 dpi CD8^+^ Tem were increased in MesLNs and remained elevated until 29 dpi (**Figures 2F, H**). At seven months *post* infection the frequencies of CD4^+^ Tem were higher than at baseline in all organs studied (**Figures 2E, F**). Frequencies of CD8^+^ Tem were increased in all organs apart from MesLNs at seven months *post* infection (**Figures 2G, H**). Antigen-specific Tem significantly increased in all organs at 8 – 12 dpi with M_2-17+_ CD4^+^ Tem being less abundant than NP_147-155^+^_ CD8^+^ Tem (**Figures 2I–L**). In MesLNs a significant increase of NP_147-155^+^_ CD8^+^ Tem was detected only at 8 dpi (**Figure 2L**). Numbers of M_2-17+_ CD4^+^ Tem increased approximately 10 – 100 times in all organs studied 8 – 29 dpi (**Figures 2M, N**). The numbers of NP_147-155^+^_ CD8^+^ Tem increased in MLNs, lungs, spleen and MesLNs at 8 dpi and remained in similar numbers in MLNs, spleen and MesLNs until 29 dpi whereas the numbers decreased in lungs (**Figures 2O, P** **)**. At seven months *post* infection frequencies and numbers of M_2-17+_ CD4^+^ Tem were increased in MLNs and ILNs whereas only the number of M_2-17+_ CD4^+^ Tem was increased in spleen (**Figures 2M, N**). Increased numbers of NP_147-155^+^_ CD8^+^ Tem were observed in lungs and in spleen at seven months *post* infection (**Figures 2O, P**). Collectively, the results are in agreement with previously published data that antigen-specific memory T cells are generated in organs directly involved in the infection. In this study, antigen-specific T cells were also detected in peripheral lymphoid organs distant from the site of viral replication. Furthermore, our data demonstrates long-term presence of antigen-specific Tem in MLN, lungs, spleen and ILNs when analyzed at seven months *post* infection.

### Lung-resident CD4^+^CD69^+^CD11a^+^ T cells increase in lung tissue after influenza virus infection

**Figure 3:**
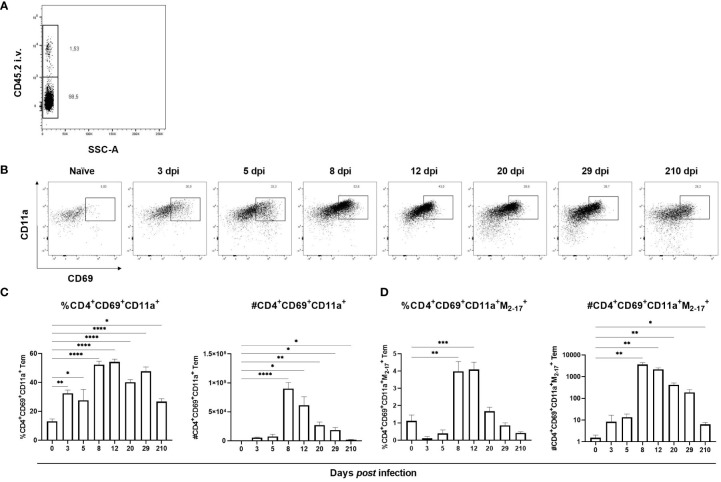
Formation of lung-resident CD4^+^ T cells in lungs after influenza virus infection. CD4^+^ Trm express CD69 and CD11a and to verify their localization to the lung tissue anti-CD45.2 antibodies were injected intravenously three minutes prior to euthanasia **(A)**. In **(B)** representative flow cytometry plots gated from CD4^+^ Tem with up to 8000 events per plot from indicated time points after infection are shown. Frequency and numbers of lung-resident CD4^+^CD69^+^CD11a^+^ T cells **(C)**. Frequency and numbers of antigen-specific, lung-resident CD4^+^CD69^+^CD11a^+^ T cells **(D)**. Data are composites of two separate experiments with five mice per group. Data are shown as mean ± SEM. *p < 0.05, **p < 0.01, ***p < 0.001 and ****p < 0.0001.

Apart from being a marker of activation, CD69 is a hallmark marker for Trm. Since an increase of CD69-expressing T cells was observed in lungs starting at 8 dpi we hypothesized that it was due to development of Trm in the lungs. In addition to CD69, CD4^+^ Trm are defined by their expression of integrin CD11a (26, 35, 36). To verify that CD4^+^CD69^+^CD11a^+^ T cells were localized to the lung tissue and not intravascularly, FITC-conjugated antibodies against the alloantigen CD45.2, present on the majority of hematopoietic cells, were injected intravenously three minutes prior to euthanasia. Indeed, >98% of the CD4^+^CD69^+^CD11a^+^ T cells were protected from staining and therefore localized in the lung tissue (**Figure 3A**). In naïve mice, CD4^+^CD69^+^CD11a^+^ T cells were scarce but from 3 dpi the frequency was significantly increased with a peak in numbers by day 8 *post* infection (**Figures 3B, C**). The frequency and number of influenza virus-specific CD4^+^ CD69^+^CD11a^+^ T cells was increased from 8 dpi until 12 – 20 dpi, and at 210 dpi the number of antigen-specific CD4^+^CD69^+^CD11a^+^ T cells remained increased (**Figure 3D**). Thus, influenza virus infection results in the development of antigen-specific, lung-resident CD4^+^CD69^+^CD11a^+^ T cells that persist at least seven months after infection.

### CD8^+^CD103^-^CD49a^+^ and CD8^+^CD103^+^CD49a^+^ lung-resident T cells dominate at different stages in lung tissue during and after influenza virus infection

**Figure 4:**
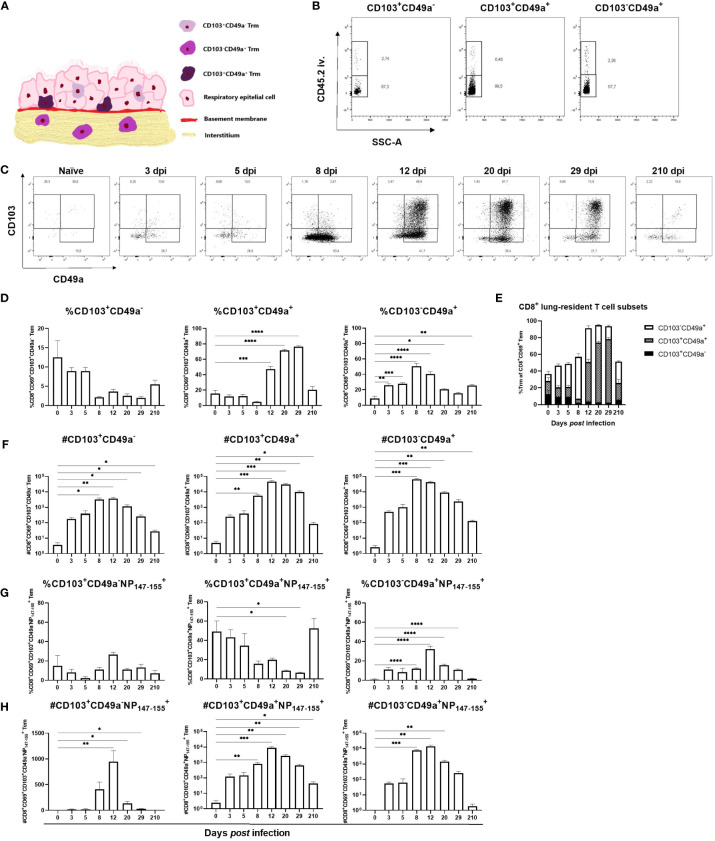
Kinetics of lung-resident CD8^+^ T cell subsets in lungs after influenza virus infection. A modified illustration of the three subsets of CD8^+^ Trm residing at different areas in the lungs as proposed in (28) **(A)**. Intravascular staining to confirm the localization of the three subsets of CD8^+^ T cells in the lung tissue **(B)**. CD103^+^CD49a^-^, CD103^-^CD49a^+^ and CD103^+^CD49a^+^ T cell populations were gated from the CD8^+^ Tem population. In **(C)**, representative flow cytometry plots from naïve mice and from influenza virus infected mice at indicated time points after infection displaying up to 8000 events. Proportion **(D)** and number **(F)** of each lung-resident CD8^+^ T cell subset at indicated time points after PR8 infection. Numbers **(H)** and frequencies **(G)** of antigen-specific, lung-resident CD8**^+^** T cell subsets. In **(E)** a summary of the findings in **(D)** is shown. Data are composites of two separate experiments with 5 – 6 mice per group. Data are shown as mean ± SEM. *p < 0.05, **p < 0.01, ***p < 0.001 and ****p < 0.0001.

Based on the proposed location of the CD8^+^ Trm subsets (**Figure 4A**) we sought to determine if differences in abundance of the different subsets could be observed at different time points after primary influenza virus infection (28). First it was verified that all three CD8^+^ subsets mainly resided in the lung tissue and not in the pulmonary vasculature (**Figure 4B**). Naïve mice had few lung-resident T cells and only a minor increase in numbers was observed until 8 dpi, coinciding with the maximum weight loss (**Figure 1A**), when the CD8^+^CD103^-^CD49a^+^ T cell subset dominated (**Figures 4C–F**). At 12 dpi the relative abundance of CD8^+^CD103^-^CD49a^+^ T cells was similar to that of CD8^+^CD103^+^CD49a^+^ T cells though slightly lower. CD8^+^CD103^+^CD49a^+^ T cells were detected at 8 dpi and peaked in numbers at 12 dpi i.e. after the influenza virus is cleared, and was the main subset at 29 dpi (**Figures 4C–F**). At seven months *post* infection, the frequency of CD8^+^CD103^-^CD49a^+^ T cells and numbers of all three lung-resident subsets remained increased (**Figures 4C, D, F**). CD8^+^CD103^+^CD49a^-^ T cells appeared to play a minor role in the response against influenza virus as this subset was represented by a low cell number and did not expand to the same extent as the other two subsets (**Figures 4C–H**). The number of antigen-specific CD8^+^CD103^+^CD49a^+^ T cells remained increased until 7 months *post* infection, but the frequency varied substantially among mice due to low cell numbers and therefore the result should be interpreted with caution. In conclusion, CD8^+^CD103^-^CD49a^+^ T cells are the dominating lung-resident CD8^+^ subset at 8 dpi whereas CD8^+^CD103^+^CD49a^+^ T cells become the most abundant subset after 12 dpi i.e. after the influenza virus has been cleared. Nevertheless, seven months after infection the frequencies of the two subsets are similar.

## Discussion

To the best of our knowledge, this study is the first to describe the kinetics of the adaptive immune response in lymphoid organs peripheral to the respiratory tract and the emergence of different lung-resident CD8^+^ T cell subsets during and after influenza virus infection. We studied the kinetics of the adaptive immune response by flow cytometry after infecting mice with PR8 intranasally and found that despite being an airway-restricted pathogen, influenza virus infection results in increased frequencies of activated B cells and T cells, and antigen-specific effector memory T cells in peripheral lymphoid organs, although to a minor extent than in tissues associated with the site of infection.

After entry, influenza virus replicates in airway-associated tissues, mainly in epithelial cells, which limits the infection to the respiratory tract. Viral antigens are taken up and transported by dendritic cells to draining MLNs within 48 hours *post* infection (13, 37–39). There, T cells recognizing cognate antigens presented on MHC by dendritic cells rapidly upregulate cell surface expression of CD69 resulting in downregulation of L-selectin and interference with sphingosine-1-phosphate receptor function to inhibit egress from the lymph node (40–42). Likewise, CD69 is upregulated on B cells shortly after activation (15). Thus, CD69 is suitable as an early activation marker of B cells and T cells. The restricted site for virus replication was mirrored by the simultaneous early activation of B cells and T cells arguing for a similar time frame for binding of the differently processed antigenic forms to the antigen-binding receptors on the respective main lymphocyte population. In line with previous findings, the proportion of CD19^+^ B cells increased in lungs from 8 dpi whereas activated CD19^+^ B cells were increased at days three and eight but also at day 12 *post* infection in our study (33). In MLNs, a peak in activated CD19^+^ B cells was detected at 3 dpi and taken together our results would be consistent with the findings that specific antibody-secreting B cells increase rapidly in MLNs only to drop close to baseline values 14 days after influenza-virus infection unlike in lungs where they increase starting from 7 dpi (43).

Although the activation of both helper T cells and cytotoxic T cells followed the same kinetic pattern in MLNs and lungs, their relative abundance was higher in lungs than in MLNs consistent with the lungs being the primary site of infection. We hypothesized that a peak of CD69-expressing T cells in MLNs would precede that of lungs since antigens are transported from lungs to MLNs to be presented to T cells followed by migration of activated, antigen-specific T cells from MLNs to lungs. In a skin infection model, infiltrating CD8^+^ T cells, but not CD4^+^ T cells, upregulate CD69 on their cell surfaces after skin entry which serves to retain the cells in the skin and later differentiate into resident memory T cells (41, 44). The increased frequencies of CD69-expressing T cells in lungs could be due to upregulation of CD69 by activated T cells migrating from MLNs and since CD69 is also associated with tissue-residency it would further argue for CD69^+^ migratory MLN T cells as the main source of activated T cells in the lungs (40). An additional explanation would be homing of peripheral, naïve T cells to the site of infection as has been demonstrated in mice infected with the intestinal nematode *Heligmosomoides polygyrus* (14, 45, 46). We observed a transient decrease in ILN cellularity which indicates a redistribution of lymphocytes to expand the population at the site of infection as previously reported in influenza virus-infected mice as well as in *Heligmosomoides polygyrus* infected mice (14, 40, 46). Virus-specific memory T cells were primarily detected in lungs and lung-draining MLNs but also in peripheral lymphoid organs not targeted by the infection. Presence of viral RNA with subsequent activation and generation of memory T cells has been observed previously in spleen after influenza virus infection but not in peripheral ILNs, MesLNs, axillary lymph nodes or brachial lymph nodes (14). Whether the increase of antigen-specific Tem in ILNs and MesLNs is due to antigen-presentation by migratory DCs from the site of infection in the same manner as in spleen or is due to migration of differentiated Tem from MLNs remains to be investigated (14, 40).

In this study, we confirmed that influenza virus infection generates lung-resident memory CD4^+^ and CD8^+^ T cells which are reported to be crucial for conveying homologous and heterosubtypic immunity that mediate a fast local protection already within 24 hours after homologous infection and by clearing the heterologous virus 36 – 48 hours faster in immune mice than in primary infected mice (18, 47, 48). Various surface markers have been identified as expressed by different subsets of tissue-resident memory T cells with CD69 as a canonical marker (29, 47, 49, 50). Expression of CD49a but not CD103 has proven to be essential for Trm to confer heterosubtypic immunity and for the cells’ ability to migrate in the tissue whereas CD103 limits migration by reducing the cells’ velocity (51). CD8^+^CD103^-^CD49a^+^ T cells expanded and was the major subset at 8 dpi coinciding with the time point when the virus is cleared (3, 33, 48) and clinical signs of infection, i.e. weight loss, are most pronounced. The ability to migrate in infected tissues is an important feature of cells involved in pathogen clearance which would support that CD8^+^CD103^-^CD49a^+^ T cells is the dominant subset initially as they do not express the speed-limiting CD103. Mice develop interstitial pneumonia after influenza virus infection and given their proposed location in the lung tissue and their effector functions as supreme perforin producers, CD8^+^CD103^-^CD49a^+^ T cells may expand to combat the infection as an effector subset rather than a memory cell subset (28, 29, 52). In influenza virus-immune mice treated with FTY720 and challenged with a heterologous virus mainly CD103^-^ tissue-resident CD4^+^ and CD8^+^ T cells conferred protective immunity indicating a possible effector role of CD8^+^CD103^-^CD49a^+^ T cells (27). However, CD49a-expression was not included in that study. Nevertheless, CD8^+^CD103^+^CD49a^+^ T cells exert similar antiviral effector functions as CD8^+^CD103^-^CD49a^+^ T cells but is the dominating subset after clearance of infection indicating a more profound memory cell phenotype and may function as sensors for new infections since immune cells in the interstitium are scarce in naïve lungs (53, 54). After cutaneous herpes simplex infection, the majority of Trm in the skin express CD49a at 7 – 9 dpi whereas at 16 dpi Trm co-express CD103 and CD49a, a phenotype that dominates in the memory phase (55). The localization of eTrm would allow them to surveil both the interstitium and epithelial cells for viral antigens enabling them to perform immunosurveillance and protect the tissue from future influenza virus infections. Thus, the presence of influenza virus-specific lung-resident T cells in the lung up to 7 months post infection is in support of such a hypothesis. Whether CD8^+^CD103^-^CD49a^+^ T cells differentiate into CD8^+^CD103^+^CD49a^+^ T cells by migrating to the basal respiratory epithelium and upregulate cell surface expression of CD103 or if the CD8^+^CD103^+^CD49a^+^ T cells develop as a separate subset remains to be investigated. In the current study, the CD8^+^CD103^+^CD49a^-^ T cells did not expand to the same extent as CD8^+^CD103^-^CD49a^+^ T cells and CD8^+^CD103^+^CD49a^+^ T cells subsets and therefore may play a minor role, if any, during influenza virus infection in mice, a theory supported by the fact that CD103^+^ Trm do not remain three months after influenza virus infection and phenotypically resemble circulating memory T cells (29).

The intranasal infection with the H1N1 influenza A virus constitutes a highly relevant model for the study of the adaptive immune response to emerging respiratory viruses including the early kinetics of differentiation of recruited, antigen-specific T cells. The results presented here increase the existing knowledge of adaptive immune responses to influenza virus infection, mapping the kinetics of tissue T cell memory expansion and contraction. In addition, we have demonstrated that two CD8^+^ lung-resident T cell subsets vary in abundance at different stages after influenza virus infection, a finding that can be further investigated in coming studies focusing on the origin, regulation, roles and functions of the respective subsets. Induction of memory T cells against heterologous influenza virus can protect the host against pandemic strains but also yields a better protection against new seasonal strains. A better understanding of the developmental steps in the kinetics of the T cell response, including Trm, would enhance the knowledge about T cell biology and aid in the generation of vaccines against respiratory viruses, in particular by targeting Trm generation and their maintenance.

## Data availability statement

The original contributions presented in the study are included in the article/**Supplementary Material**. Further inquiries can be directed to the corresponding author.

## Ethics statement

The animal study was reviewed and approved by the regional animal ethics committee of Uppsala, Sweden.

## Author contributions

ME and K-OG designed the study, wrote the manuscript, collected data and interpreted the results. ME performed the *in vivo* experiments, performed the data analysis and prepared the figures. ME, SN and K-OG reviewed, discussed and approved the final version of the manuscript. All authors contributed to the article and approved the submitted version.

## Funding

This work was funded by the National Veterinary Institute and Uppsala Immunobiology Lab. The National Veterinary Institute funded ME, the animal studies and reagents. Uppsala Immunobiology Lab funded reagents and the research lab. SN and publishing cost were supported by Karolinska Institute intramural funds.

## Acknowledgments

We thank the NIH Tetramer Core Facility (contract number 75N93020D00005) for providing the tetramers.

## Conflict of interest

The authors declare that the research was conducted in the absence of any commercial or financial relationships that could be construed as a potential conflict of interest.

## Publisher’s note

All claims expressed in this article are solely those of the authors and do not necessarily represent those of their affiliated organizations, or those of the publisher, the editors and the reviewers. Any product that may be evaluated in this article, or claim that may be made by its manufacturer, is not guaranteed or endorsed by the publisher.
