# Supplementary material for: T cell kinetics reveal expansion of distinct lung T cell subsets in acute versus in resolved influenza virus infection
Source: Front Immunol. 2022 Oct 6;13:949299. doi: 10.3389/fimmu.2022.949299 (PMC9582761; doi:10.3389/fimmu.2022.949299)
Supplement: Supplementary file 1 [file DataSheet_1.docx]

Supplementary Material

| Group | MLNs | Lungs | Spleen | ILNs | Bone marrow |
| --- | --- | --- | --- | --- | --- |
| 10^4^ TCID_50_ PR8 | 26.77 | 21.67 | nd | nd | nd |
| 10^4^ TCID_50_ PR8 | 22.33 | 21.67 | nd | nd | nd |
| 10^4^ TCID_50_ PR8 | 25.29 | 21.81 | nd | nd | nd |
| Mean ± SD | 24.80 ± 2.26 | 21.27 ± 0.08 | nd | nd | nd |
|  |  |  |  |  |  |
| 10^6^ TCID_50_ PR8 | 18.15 | 18.76 | nd | nd | nd |
| 10^6^ TCID_50_ PR8 | 22.87 | 17.07 | nd | nd | nd |
| 10^6^ TCID_50_ PR8 | 20.10 | 18.06 | nd | nd | nd |
| Mean ± SD | 20.37 ± 2.37 | 17.96 ± 0.85 | nd | nd | nd |
|  |  |  |  |  |  |
| Positive control: 21.9 |  |  |  |  |  |
| Negative control: nd |  |  |  |  |  |

**Supplementary Table 1: Influenza virus replication is restricted to airway-associated tissues**

Examination by RT-PCR for presence of viral RNA in tissues from PR8-infected mice. Groups of three naïve BALB/c mice were infected by intranasal route with 10^4^ TCID_50_ PR8 or 10^6^ TCID_50_ PR8. At 48 hours *post* infection the mice were sacrificed and individual tissue specimens were examined with RT-PCR for the presence of viral RNA. Values indicate mean Ct value ± standard deviation (SD), nd = not detected.

The standard curve below shows Ct values at different titers of virus (TCID_50_).


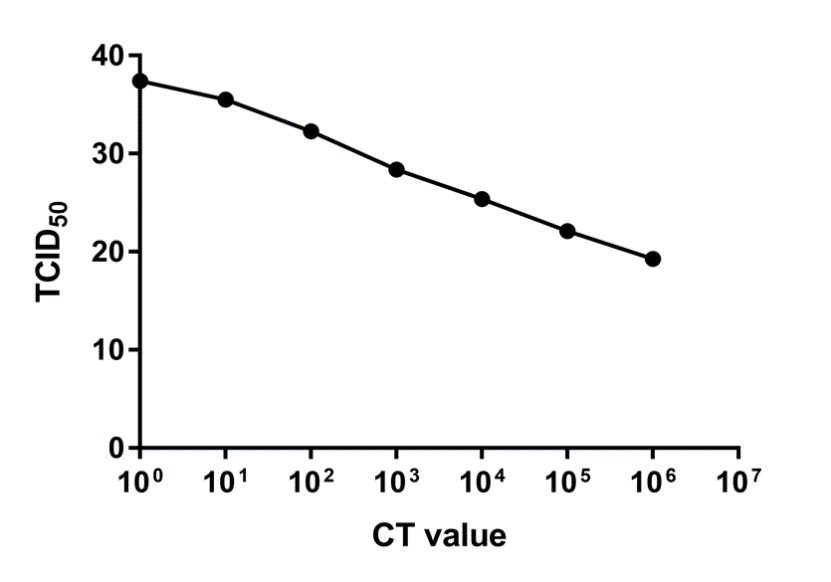

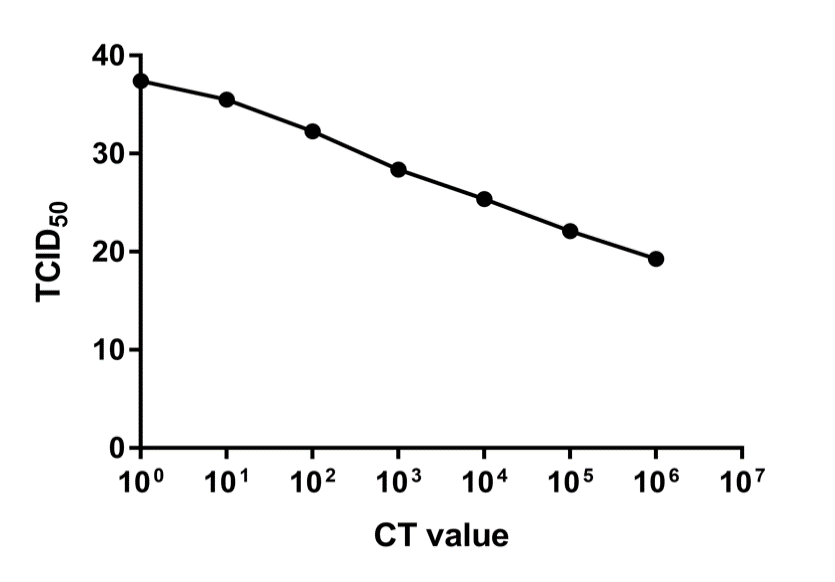

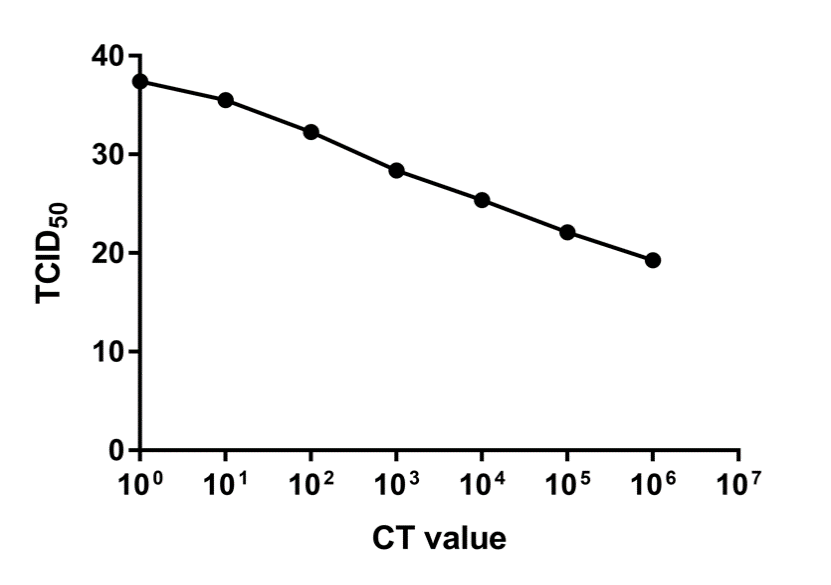


**
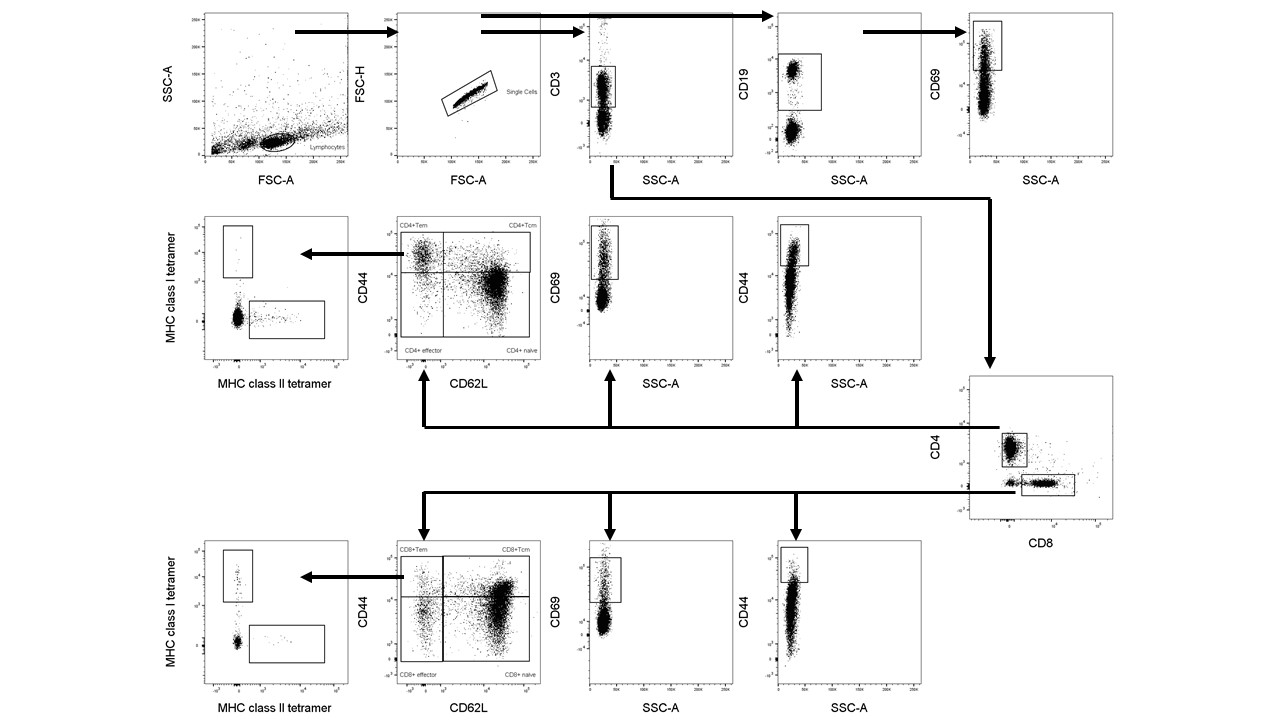
**

**Supplementary Figure 1.** Gating strategy for activated T and B cells, and CD44^hi^ and Tem. Up to 10000 events are displayed. FMO-controls were used to define gates. CLIP-loaded tetramers were used as a negative control for class I and class II MHC tetramers.


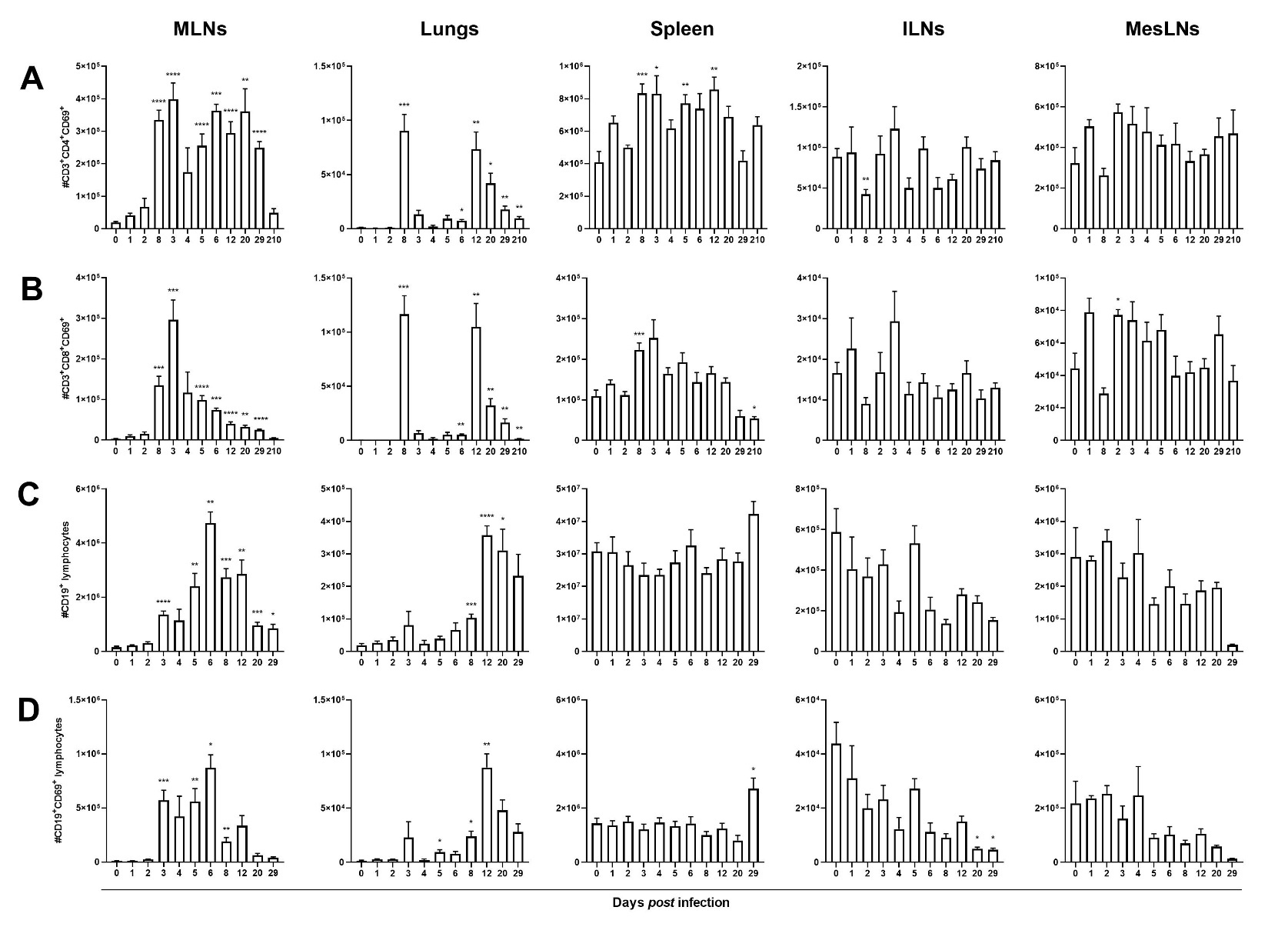


**Supplementary Figure 2.** Absolute numbers of CD4^+^CD69^+^ T cells **(A)**, CD8^+^CD69^+^ T cells **(B)**, CD19^+^ B cells **(C)** and CD19^+^CD69^+^ cells **(D)** in MLNs, lungs, spleen, ILNs and MesLNs. Data in figures are composites of 1 – 4 separate experiments per timepoint with 3 – 10 mice per group. Data are shown as mean ± SEM. * = p< 0.05, ** = p< 0.01, *** = p< 0.001 and **** = p< 0.0001.


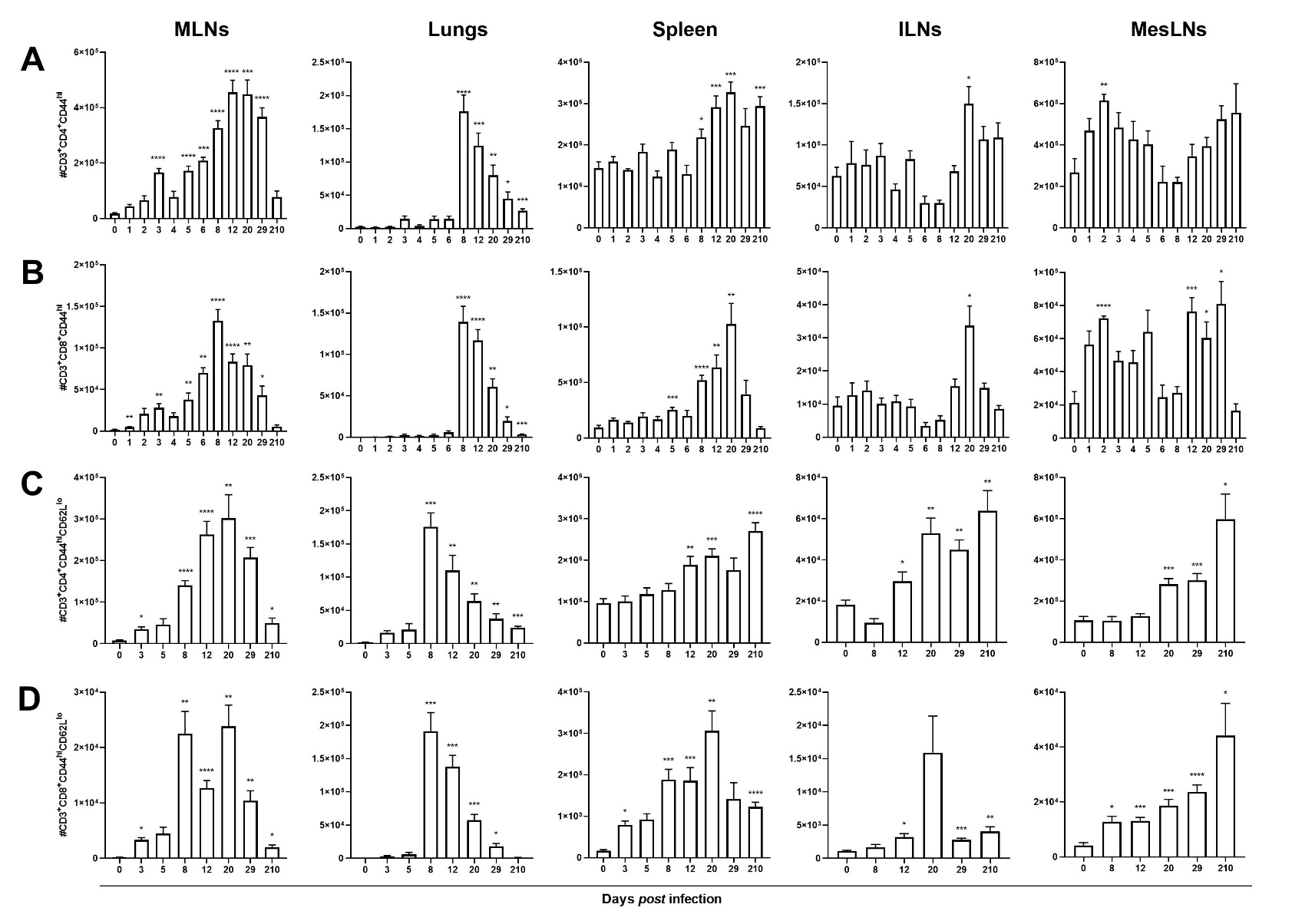


**Supplementary Figure 3.** Absolute numbers of CD4^+^CD44^hi^ T cells **(A)**, CD8^+^CD44^hi^ T cells **(B)**, CD4^+^ Tem **(C)** and CD8^+^ Tem **(D)** in MLNs, lungs, spleen, ILNs and MesLNs. Data in figures A and B are composites of 1 – 4 separate experiments per timepoint with 3 – 10 mice per group. Data in figures C and D are composites of 2 – 3 separate experiments per timepoint with 4 – 10 mice per group. Data are shown as mean ± SEM. * = p< 0.05, ** = p< 0.01, *** = p< 0.001 and **** = p< 0.0001.
